# The Impact of Entrepreneurial Passion on Psychology and Behavior of Entrepreneurs

**DOI:** 10.3389/fpsyg.2020.01733

**Published:** 2020-07-21

**Authors:** Bing Feng, Min Chen

**Affiliations:** ^1^School of Economics and Management, Northwest University, Xi’an, China; ^2^School of Management, Yulin University, Yulin, China; ^3^Academy of Financial Research, School of Business, Wenzhou University, Wenzhou, China

**Keywords:** entrepreneurial passion, self-efficiency, entrepreneurial persistence behavior, psychology, enterprise performance, entrepreneurial persistence

## Abstract

In order to study the influence of entrepreneurial passion on entrepreneurs’ psychology and behavior, based on the theory of self-efficacy, a model of relationship between entrepreneurial passion and entrepreneurs’ psychology and behavior was constructed, relevant hypotheses were proposed, and the promotion mechanism of entrepreneurial passion on entrepreneurial behavior and enterprise performance was analyzed. A survey of 300 entrepreneurs from Hangzhou, Wenzhou, Jiaxing, Shaoxing, and Huzhou was conducted to verify the reliability of the questionnaire through statistical description and analysis. Then exploratory factor analysis and confirmatory factor analysis (CFA) were conducted to test the correlation between variables. Finally, the structural equation was simulated to verify the correctness of the proposed hypothesis and model. The results show that the designed questionnaire has good reliability [the correction item total correlation coefficients (CITC) of all scales are greater than 0.3, values of Cronbach’s α are higher than 0.6], the validity (all inventory accumulation explanation degree are higher than 50%) and the fitting (χ^2^/df values of all scales are less than 3, comparative fitness index (CFI), goodness of fitness index (GFI), and incremental fitness index (IFI) are greater than 0.9, root-mean-square error of approximation (RMSEA) value is less than 0.08). The direct effect of harmonious passion on entrepreneurial persistence and enterprise performance is not significant, while the direct effect of compulsive passion on entrepreneurial persistence and enterprise performance is significant. Harmonious passion (*P* < 0.001) and compulsive passion (*P* < 0.01) are significantly correlated with entrepreneurial self-efficacy, and self-efficacy plays a mediating role between entrepreneurial passion and entrepreneur psychology and behavior (*P* < 0.05), and the hypothesis proposed is basically valid. Therefore, entrepreneurial passion can positively guide the entrepreneurial persistence of entrepreneurs, and at the same time promote the performance of enterprises by stimulating the positive emotions of entrepreneurs. In addition, entrepreneurs can enhance their entrepreneurial role identity, maintain a positive attitude, stimulate creativity, and innovation, to enhance their sense of energy efficiency. The government can also promote successful business cases to build an inclusive and innovative social environment and stimulate the entrepreneurial passion of entrepreneurs. This study reveals the relationship between entrepreneurial passion, self-efficacy, and entrepreneur psychology and behavior, and extends the application of entrepreneurial passion in the field of entrepreneurship.

## Introduction

With the development and progress of economic globalization, more and more people choose to start their own businesses under strong national and governmental supports, which not only promotes the economic development of the country, but also alleviates the employment situation to a certain extent (Barba-Sánchez and Atienza-Sahuquillo, 2018). According to relevant research, an increasing number of office workers and college students are involved in the army of self-entrepreneurship, and entrepreneurship has become a trend in China (Azoulay et al., 2020). However, some enterprises cannot continue to operate after establishment, and the entrepreneurial success rate is low. This is because when entrepreneurs encounter difficulties and setbacks, only a part of them can continue to start a business (Bosma et al., 2018; Acs et al., 2018). To explain the psychology and behavior of entrepreneurs in depth, more and more researchers are continuously enriching and developing theories related to entrepreneurship.

In recent years, the development of positive psychology has attracted the attention of many scholars, and passion, as an important theory of positive psychology, has a profound impact on people’s psychological and behavioral activities (Stroe et al., 2018). Currently, researchers have used passion theory to explain entrepreneurial behavior. Entrepreneurial passion is a core trait that entrepreneurs must possess, which can encourage entrepreneurs to conduct entrepreneurial behaviors. When entrepreneurs face difficulties, entrepreneurial passion can be used as a support force to keep them going (Costa et al., 2018; Montiel-Campos, 2018). By persisting in entrepreneurial behavior, relentlessly pursuing established goals, and investing a lot of time and energy, entrepreneurs can achieve successful entrepreneurship and obtain economic benefits (Milanesi, 2018). Therefore, it is of theoretical and practical significance to study the influence of entrepreneurial passion on the psychology and behavior of entrepreneurs.

Based on this, in this research, the influence of entrepreneurial passion on the psychology and behavior of entrepreneurs was explored and a model of the relationship between entrepreneurial passion and the psychology and behavior of entrepreneurs was constructed according to the theories of entrepreneurial passion and self-efficacy (Deleuze et al., 2018). In addition, through the questionnaire survey, the promotion mechanism of entrepreneurial passion on entrepreneurial behavior and enterprise performance was discussed, and the reliability of the questionnaire was analyzed. It is hoped that this study can reveal the relationship between entrepreneurial passion, self-efficacy, and psychology and behavior of entrepreneur, and expand the application of entrepreneurial passion in the field of entrepreneurship.

## Literature Review

Costin et al. (2019) studied the influence of simulation games in the development of cognitive (knowledge and skills) and non-cognitive (attitude) entrepreneurial capabilities using the existing entrepreneurial capability framework. The benefits of using business simulation as an effective model for developing entrepreneurial capabilities were demonstrated. This ability was not only beneficial to individuals working in entrepreneurial environments, but can be transferred to any business environment, thus highlighting the importance of entrepreneurial learning for all students (Costin et al., 2019). Cannatelli et al. (2019) explored the relationship between the alternative entrepreneurial behavioral logic based on the principle of causality and the two passions, namely the passion for product and the passion for growth. The results showed that entrepreneurs who were passionate about products prioritized decisions based on implementation principles, while those who were passionate about growth relied primarily on causal logic. Moreover, this relationship would indeed influence the direction they pursued in their career, which was beneficial to homogeneous and heterogeneous strategies (Cannatelli et al., 2019).

Bao et al. (2017) studied the relationship between entrepreneurial passion, opportunity recognition, and entrepreneurial behavior. Entrepreneurial enthusiasm included the strong positive emotions generated by participation in entrepreneurial activities and the central role of these activities in the entrepreneur’s self-identity. The results showed that entrepreneurial enthusiasm had an important influence on opportunity recognition and entrepreneurial behavior, and opportunity recognition partly mediated the relationship between entrepreneurial enthusiasm and entrepreneurial behavior. Therefore, entrepreneurs with enthusiasm were more likely than others to find opportunities and start new businesses (Bao et al., 2017). Chen and Zhou (2017) studied the importance of entrepreneurs’ internal and external social capital in the relationship between entrepreneurs’ self-efficacy (ESE) and enterprises’ innovation behaviors, and analyzed data from 193 Chinese entrepreneurs. The results showed that ESE had a positive impact on the innovation behavior of the company, while the internal social capital of the entrepreneur played a negative mediating role in the relationship between ESE and the innovation behavior of the company (Chen and Zhou, 2017).

According to relevant domestic and foreign researches, it is found that the researches on entrepreneurial passion, entrepreneurial behavior, and self-efficacy are developing rapidly at present. However, there is still a lack of corresponding researches on the relationship between entrepreneurial passion, self-efficacy, and entrepreneurial behavior from the perspective of psychology. In the study, based on the entrepreneurial passion and self-efficacy theory, the influence of entrepreneurial passion on the psychology and behavior of entrepreneurs was discussed, the relationship model between entrepreneurial passion and the psychology and behavior of entrepreneurs was constructed, and the promotion mechanism of entrepreneurial passion on entrepreneurial behavior and enterprise performance was analyzed through questionnaire survey.

## Materials and Methods

### Entrepreneurial Passion and Self-Efficiency Theory

At present, researchers have different opinions on the definition of entrepreneurial passion, which mainly focus on three aspects: individual trait perspective, emotion perspective, and motivation perspective.

First, the perspective of individual trait is an innate personality characteristic of individuals, which makes individuals have different characteristics from others in different situations and stabilize their existence. However, with the establishment and development of enterprises, most entrepreneurs will lose their entrepreneurial passion and cannot be explained by individual characteristics (Yu et al., 2019).

Second, the emotional perspective, including the five psychological states of feeling, cognition, expression, physiology, and action, is the psychological and physiological response triggered by the stimulation of the external environment. Researchers believe that entrepreneurial passion is an emotional experience, not a reflection of individual characteristics. When entrepreneurs have abundant entrepreneurial passion, they can do the entrepreneurial behavior in line with their identity. With the in-depth study of the theory of entrepreneurial passion, the explanation of entrepreneurial passion from this perspective has been recognized by people.

Third, the motivation perspective is the driving force that motivates individuals to achieve their goals and carry out corresponding activities. Passion is a key part of the motivation to start a business and can make an individual work harder. Under specific motivation, entrepreneurial passion can stimulate the entrepreneur’s thinking and act accordingly.

According to the internalization of passion activity in individual identity, passion is divided into harmonious passion (HP) and obsessive passion. Harmonious passion is that individuals independently choose the activities they like, generate positive emotions, and get a fuller experience. And obsessive passion refers to the negative emotions caused by the passive pressure of individuals when they participate in their favorite activities. The differences between the two are as follows. First, HP is more flexible and harmonious than obsessive passion, and tends to produce more positive emotions. Second, HP is more effective than obsessive passion in making people stick to an activity (Zheng and Liu, 2020). When people find that they can benefit from the activity, they will stick to the activity. Conversely, if negative emotions are often obtained, people will reduce or even stop the activity. Third, in a competitive environment, obsessive passion has an advantage over HP.

In the study of the antecedent variable of entrepreneurial passion, that is, how to generate entrepreneurial passion, it is currently believed that its source includes two antecedent variables: identity and entrepreneurial effort. Some researchers believe that entrepreneurial passion is closely related to the identity of entrepreneurs, which is a self-concept and a guide to individual actions, including significance and central identity analysis, and it is a complex entity. According to the self-regulation theory, entrepreneurial efforts will make entrepreneurs develop positive emotions. When entrepreneurs take voluntary actions, they can judge their own emotions accordingly, and entrepreneurial efforts will cause changes in entrepreneurial passion.

In the theory of motivation, it is believed that the behavior of individuals is affected by the motivation. In the field of psychology, motivation is the psychological tendency to initiate and maintain an individual’s behavior and make the behavior develop toward a specific goal (Chen, 2018). Self-efficacy is an individual’s subjective judgment of his/her ability to accomplish a specific goal in a specific situation, including two parts: outcome expectation and efficacy expectation. Self-efficacy is not the skill itself that an individual possesses, but the degree of confidence that an individual can achieve a given goal by performing a certain behavior to himself. In different fields, self-efficacy varies according to an individual’s abilities and skills. Entrepreneurial self-efficacy is the self-confidence manifestation of entrepreneurs’ success in entrepreneurial behavior and their own capabilities. The higher the entrepreneurial self-efficacy, the more confident the entrepreneur is that they can influence the surrounding environment through their own abilities. Entrepreneurial self-efficacy can predict entrepreneurial psychology and behavior, representing the entrepreneur’s ability to complete the assessment and perception of entrepreneurial behavior.

### The Influence of Entrepreneurial Passion on the Psychology and Behavior of Entrepreneurs

With the development of positive psychology, researchers gradually combine it with entrepreneurship to study its importance. As the product of the combination of positive psychology and entrepreneurship, entrepreneurship passion has been found to promote the entrepreneurial behavior, entrepreneurial efforts, and other behaviors and psychology of entrepreneurs. For entrepreneurs, their entrepreneurial goal is to improve enterprise performance and achieve entrepreneurial success. Entrepreneurial passion has an impact on enterprise performance through psychology. The role identity of the entrepreneur originates from the positive intrinsic affirmation of the entrepreneur’s real self-concept, so the entrepreneurial passion will be related to the development of the role identity. When a particular identity is activated, the experience of entrepreneurial passion mobilizes the entrepreneur’s self-regulatory process to pursue the entrepreneurial goals that match it. At the same time, the participation in cognitive activities also validates this particular focused identity, which in a sense is a characteristic of strong positive emotions.

First, from the organizational point of view, entrepreneurial passion can affect the establishment of enterprises and the effective realization of goals. Compared with individuals lacking entrepreneurial passion, entrepreneurs with passion are more willing to promote the development of enterprises. Therefore, entrepreneurial passion has a positive impact on the development and growth of enterprises and can effectively predict the growth of enterprises. When driven by entrepreneurial passion, entrepreneurs can more effectively identify entrepreneurial opportunities and create and nurture business development. When the entrepreneurial passion is at a lower level, the entrepreneurs will spend much less time and energy on entrepreneurship. When it is below a certain level, they will even withdraw from entrepreneurial activities. However, when the entrepreneurial passion of entrepreneurs is at a high level, entrepreneurs will be in a state of overconfidence in their entrepreneurial ideas and concepts, which may lead to high expectations and have a negative effect on the growth of enterprises.

Second, from the perspective of individuals, entrepreneurial passion can have a positive and significant impact on individuals. Entrepreneurial passion will generate an internal driving force for individual behavior, so as to better improve individual performance and benefit individuals to obtain material gains. If entrepreneurs are full of entrepreneurial passion, they will have a sense of enjoyment in the process of starting a business, so that they will devote more time and energy to entrepreneurial activities, so as to have a higher degree of satisfaction with entrepreneurial activities. Entrepreneurial passion will also have a direct and indirect impact on entrepreneurs’ behavior, which is a series of activities related to the establishment and operation of enterprises.

The direct impacts are introduced as follows: entrepreneurial passion will stimulate the entrepreneurial behavior of entrepreneurs and bring the excitement of pursuing goals. Therefore, entrepreneurial passion and entrepreneurial persistence behavior have a positive correlation. At the same time, the higher the level of HP, the more significant the relationship with persistence. This is because HP stimulates the driving force to promote entrepreneurial behavior.

The indirect effects are introduced as follows: entrepreneurial passion also influences entrepreneurial behavior through certain intermediations, such as cognitive processes and intrinsic motivation. Studies have shown that emotions influence entrepreneurs’ behavior by affecting cognitive processes. When entrepreneurs with high entrepreneurial passion are confronted with adverse market information, their sensitivity is reduced and they stick to their previous behavior. Entrepreneurial passion provides entrepreneurial behavior motivation for entrepreneurs. Similarly, individuals with passionate experiences are often able to feel intrinsic motivation for entrepreneurial behavior.

### Model Construction and Theoretical Hypothesis

**FIGURE 1:**
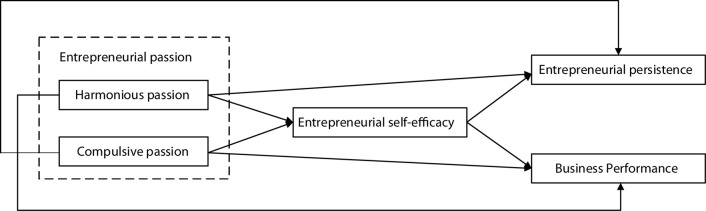
The relationship model between entrepreneurial passion, entrepreneurial self-efficacy, entrepreneurial persistence behavior, and enterprise performance.

According to the theory of motivational self-determination, HP comes from autonomous internalization, individuals can obtain positive emotional experience, while obsessive passion comes from controlled internalization, which is affected by pressure, self-worth, etc. In highly competitive environments, however, obsessive passion can have a positive effect on entrepreneurs. At the same time, the relationship between entrepreneurial passion and entrepreneurial behavior will be affected by self-efficacy. In this research, the relationship model of entrepreneurial passion, entrepreneurial self-efficacy, entrepreneurial persistence behavior, and enterprise performance was constructed, as shown in Figure 1.

First, the relationship between entrepreneurial passion, entrepreneurial persistence behavior, and enterprise performance.

Entrepreneurial passion is the strong psychological emotion that entrepreneurs show in the process of starting a business, and they have a positive tendency toward entrepreneurial behavior and pay a lot of energy and time for it. When entrepreneurs agree with the value of the target they pursue, they will stick to the pursuit of the target even if they lack the corresponding ability and skills. Entrepreneurial passion belongs to positive emotional experience, which also indicates that things are going well and there is no need to reevaluate and change individual behaviors. Individuals with positive experiences choose to stick to their current behavior to maintain this state, thereby gaining enterprise performance.

For the HP, the individual can control the behavior activities independently and flexibly. As long as the positive benefits can be obtained, the individual will stick to the behavior activities. However, obsessive passion makes entrepreneurs lack independent control and flexibility, and under the influence of external environmental factors, entrepreneurs will have stubborn and persistent behavior toward entrepreneurial activities. Based on this, the following hypotheses were proposed.

H1: entrepreneurial passion has a positive impact on entrepreneurial persistence behavior and enterprise performance.

H1-1: HP has a positive impact on entrepreneurial persistence behavior and enterprise performance.

H1-2: obsessive passion has a positive impact on entrepreneurial persistence behavior and enterprise performance.

Second, the impact of entrepreneurial passion on self-efficacy.

Self-efficacy is the confidence of entrepreneurs in their own ability in the process of starting a business, which can be strengthened through individual assessment. Negative emotions can bring bad feelings to individuals, while positive emotions can trigger the confidence of entrepreneurs in the process of entrepreneurial success. Entrepreneurs with HP have the ability to choose independently and bring positive emotional experience. Entrepreneurs with obsessive passion may bring more negative emotions because they lack the right to choose independently. However, entrepreneurs with obsessive passion in a highly competitive environment will have stronger psychological adjustment ability than HP, thus producing positive emotions. Accordingly, entrepreneurs may actively learn corresponding skills and abilities to improve self-efficacy. The higher the entrepreneur’s passion, the higher the self-efficacy. Based on this, the following hypotheses were proposed.

H2: entrepreneurial passion has a positive effect on self-efficacy.

H2-1: HP has a positive effect on self-efficacy.

H2-2: obsessive passion has a positive effect on self-efficacy.

Third, the influence of self-efficacy on entrepreneurial persistence behavior and enterprise performance.

In addition to passion, entrepreneurial self-efficacy is also an important factor affecting the behavior of entrepreneurs. Self-efficacy can distinguish the difference of psychological characteristics between entrepreneurs and non-entrepreneurs and predict the entrepreneurial behavior and entrepreneurial tendency of entrepreneurs. Under certain circumstances, high self-efficacy can motivate individuals to make more efforts voluntarily for the set goals, thus having high persistence. While low self-efficacy lacks this effect. Therefore, high self-efficacy can make entrepreneurs persist in entrepreneurial behavior for a long time and further promote enterprise performance. Based on this, the following hypotheses were proposed.

H3: self-efficacy has a positive impact on entrepreneurial persistence behavior and enterprise performance.

Fourth, the relationship between entrepreneurial passion, self-efficacy, entrepreneurial persistence behavior, and enterprise performance.

The negative emotional experience of an individual in a complex environment is affected by self-efficacy, and his own way of thinking will affect the emotional response, so as to affect the behavior. Entrepreneurs with high self-efficacy will make more efforts to cope with difficulties, while entrepreneurs with low self-efficacy may reduce their efforts or even give up their current entrepreneurial behaviors. When entrepreneurs full of entrepreneurial passion are confident in their own abilities and believe that the current entrepreneurial behavior can bring high enterprise performance, entrepreneurs will show high entrepreneurial persistence behavior. Based on this, the following hypotheses were proposed.

H4: self-efficacy mediates between entrepreneurial passion, entrepreneurial persistence behavior, and enterprise performance.

H4-1: self-efficacy mediates between HP, entrepreneurial persistence behavior, and enterprise performance.

H4-2: self-efficacy mediates between obsessive passion, entrepreneurial persistence behavior, and enterprise performance.

### Questionnaire Design and Research Samples

In this study, the model and hypothesis were empirically analyzed by means of questionnaire survey, and all variables were measured by relatively mature and widely used scales at home and abroad (Chen, 2019).

For entrepreneurial passion, the measurement scale of Anjum et al. (2018) was adopted, and the scale was translated. Among them, the entrepreneurial passion contains 14 items, the HP and the obsessive passion each contain 7 items. Besides, Likert scale with level 1–5 was adopted, in which1 means “completely disagree,” and 5 means “completely agree.” The complete questionnaire is shown in Table 1.

**TABLE 1:** Entrepreneurial passion questionnaire.

No.	Items	1	2	3	4	5
1	In the process of starting a business, I get a sense of identity					
2	The harvest in the process of starting a business makes me like it more					
3	Starting a business doesn’t affect my daily life					
4	Entrepreneurship enriches my personal experience					
5	Entrepreneurship is an uncontrollable passion					
6	I can devote myself to entrepreneurial activities					
7	Entrepreneurship is a memorable experience					
8	I am so eager to start a business that I couldn’t help but get involved					
9	Without entrepreneurship, I can’t imagine what kind of life I would have					
10	Starting a business is part of my life					
11	Entrepreneurial activities affect my personal emotions					
12	I can’t help myself from starting a business					
13	I’m obsessed with entrepreneurship					
14	Whether or not I can start my own business successfully determines my personal mood					

For self-efficacy, entrepreneurial self-efficacy scale of Brändle et al. (2018) was adopted. There were 15 questions, including entrepreneurs’ entrepreneurial self-efficacy in innovation, marketing, finance, and other aspects. Besides, Likert scale with level 1–5 was adopted, as shown in Table 2.

**TABLE 2:** Questionnaire of entrepreneurial self-efficacy.

No.	Items	1	2	3	4	5
1	Innovation ability					
2	Financial analysis ability					
3	Industry understanding					
4	Ability to explore new markets					
5	Ability to develop and implement sales plan					
6	Ability to design new products and services					
7	Ability to reduce risk					
8	Ability to implement strategic planning					
9	Ability to establish product market position					
10	Ability to define management and accountability					
11	Ability to take risks					
12	Ability to establish strategic goals and tasks					
13	Ability to propose new methods of production, marketing, and management					
14	Ability to develop systems and internal controls					
15	Ability to make decisions at risk					

For entrepreneurial persistence behavior, a measurement scale of D’arc da Silva Brito et al. (2018) was used, with a total of 6 items, and Likert scale with level 1–5was adopted, as shown in Table 3.

**TABLE 3:** Entrepreneurial persistence behavior questionnaire.

No.	Items	1	2	3	4	5
1	When others give up entrepreneurship, I keep going					
2	When others oppose me starting a business, I still stick with it					
3	Whenever I encounter any difficulties or setbacks, I insist on starting a business					
4	Entrepreneurship increases my life satisfaction					
5	When starting a business, I often suspend work at hand to perform other duties					
6	I put more effort into starting a business than anyone else					

For enterprise performance, the measurement scale of Lawton et al. (2019) was used. There were 6 items in total and Likert scale with level 1–5 was adopted, as shown in Table 4.

**TABLE 4:** Enterprise performance questionnaire.

No.	Items	1	2	3	4	5
1	Whether the sales volume and growth rate reach the expected target?					
2	Whether the corresponding gross profit is obtained?					
3	Are products and services innovative?					
4	Whether the cost is within the expected control range?					
5	Whether the market share of the product is increasing?					
6	Whether the customer is satisfied with the product or service?					

The entrepreneur’s entrepreneurial persistence behavior is influenced by the entrepreneur’s gender, work experience, previous entrepreneurial experience, and other factors, so these factors are taken as control variables for data analysis.

**FIGURE 2:**
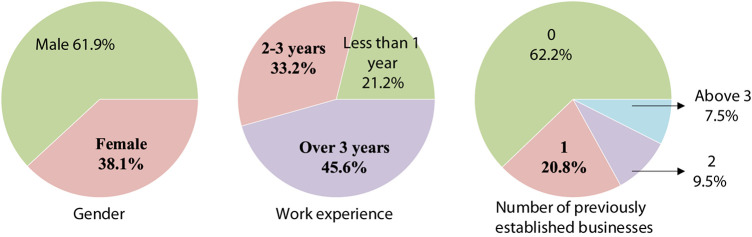
Sample statistical distribution results.

The respondents of this study mainly come from entrepreneurs in Hangzhou, Wenzhou, Jiaxing, Shaoxing, and Huzhou, whose enterprises have been in business for less than 8 years, and the survey period is from May 2019 to October 2019. In this study, a total of 300 questionnaires were distributed, and 263 questionnaires were recovered. The unqualified questionnaires were removed, and 226 questionnaires were effectively recovered. By analyzing the basic information of the questionnaire sample, the authors obtained the statistical distribution results of entrepreneur gender, work experience, and previous entrepreneurship experience, as shown in Figure 2.

### Statistical Methods

In this study, software SPSS22.0 and software AMOS20.0were used for data analysis. Items analyzed included: I. Conducting descriptive statistics and correlation analysis. II. The reliability and validity of the questionnaire are verified by exploratory factor analysis and factor analysis. III. Through the structural equation AMOS20.0, the correlation between self-efficacy, entrepreneurial passion and entrepreneur persistence and enterprise performance is studied.

## Results

### Questionnaire Reliability

Reliability was used to analyze the reliability and stability of the results and to reflect the accuracy of the questionnaire data. In this study, Cronbach α coefficient (Lawton et al., 2019) and total correlation coefficient of corrected term CITC (Hong et al., 2018) were used to test the reliability of variables. It is generally believed that the value of Cronbach’s α coefficient is between 0 and 1. If the value is less than 0.6, the reliability is low and the questionnaire needs to be written again. If the value is between 0.7 and 0.8, it indicates a certain degree of reliability. If it exceeds 0.8, the reliability is very good. The CITC value is bounded by 0.3, and the Cronbach’s α value lower than 0.3 or the deleted item can be increased.

**FIGURE 3:**
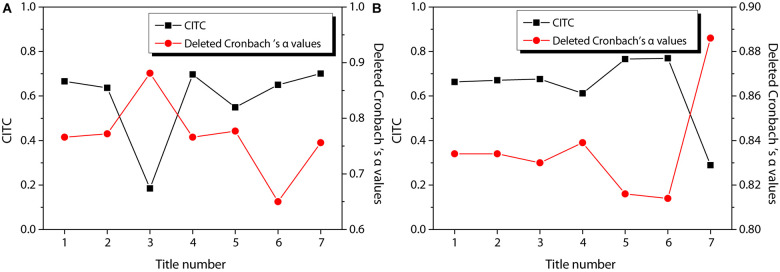
Reliability test of entrepreneurial passion. **(A)** harmonious passion; **(B)** obsessive passion.

**FIGURE 4:**
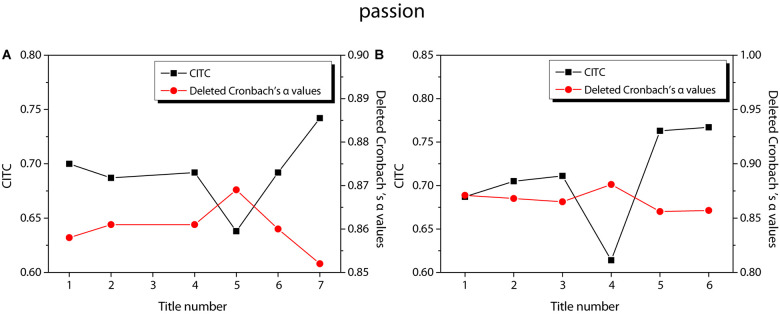
Re-reliability test of entrepreneurial passion. **(A)** harmonious passion; **(B)** obsessive passion.

Figure 3 shows the reliability test results of entrepreneurial passion, in which Figure 3A shows the reliability test of HP and Figure 3B shows the reliability test of obsessive passion. According to the figure, the CITC value of item 3 of HP is lower than 0.3, and the reliability coefficient is 0.881 after the item is deleted. The CITC value of item 7 of obsessive passion is lower than 0.3, and the reliability coefficient is 0.886 after the item is deleted. Therefore, these two questions are deleted and the reliability test is performed again. The results are shown in Figure 4. The CITC values obtained after deletion are all higher than 0.3. If any item is deleted, its reliability coefficient is lower than the existing reliability coefficient value, indicating that these items need to be retained.

**FIGURE 5:**
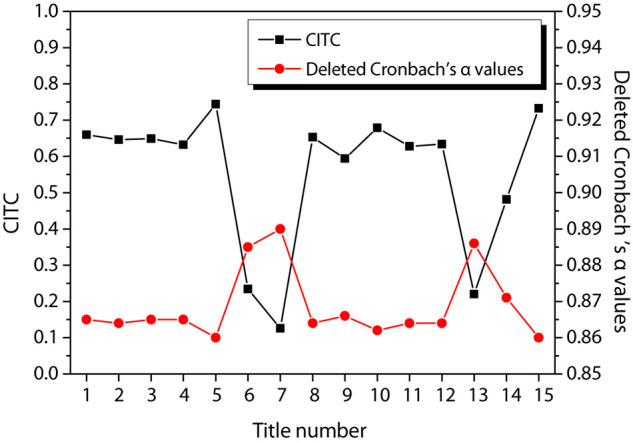
Reliability test of entrepreneurial self-efficacy.

**FIGURE 6:**
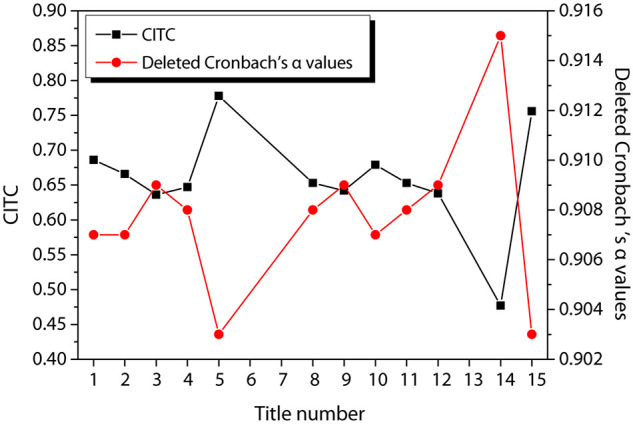
Re-reliability test of entrepreneurial self-efficacy.

Figure 5 shows the reliability test results of entrepreneurial self-efficacy. As can be observed from the figure, the values of Cronbach’s α coefficients are all higher than 0.8 and have good reliability, but the CITC values of items 6, 7, and 13 are all less than 0.3. After these items are deleted, their reliability coefficients are 0.885, 0.890, and 0.886, respectively. After the reliability test is performed again, the results are shown in Figure 6. The CITC value of all items after deleting a certain item is higher than 0.3. After any item is deleted, its reliability coefficient is lower than the value of the existing reliability coefficient, indicating that these items need to be retained.

**FIGURE 7:**
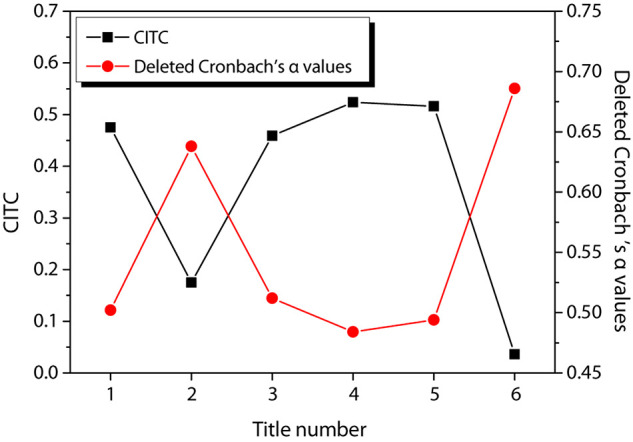
Reliability test of entrepreneurial persistence behavior.

**FIGURE 8:**
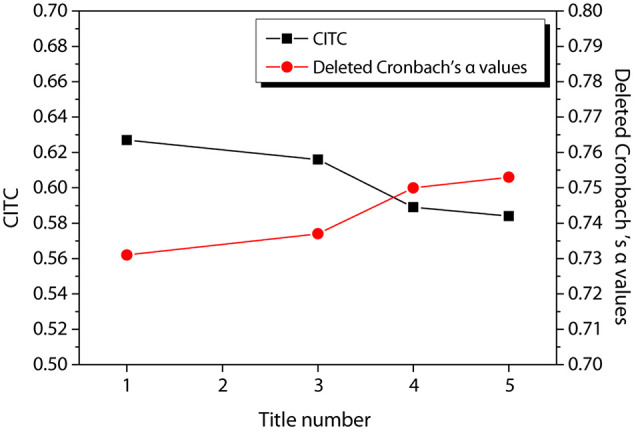
Re-reliability test of entrepreneurial persistence behavior.

Figure 7 shows the reliability test results of entrepreneurial persistence behavior. As can be observed from the figure, the CITC values of items 2 and 6 of entrepreneurial persistence behavior are all less than 0.3. After these items are deleted, their reliability coefficients are 0.637 and 0.689, respectively. Therefore, these two items are deleted respectively and reliability test is conducted again. The results are shown in Figure 8. The CITC values obtained after deleting a certain item are all higher than 0.3. After any item is deleted, its reliability coefficient is lower than the value of the existing reliability coefficient, indicating that these items need to be retained.

**FIGURE 9:**
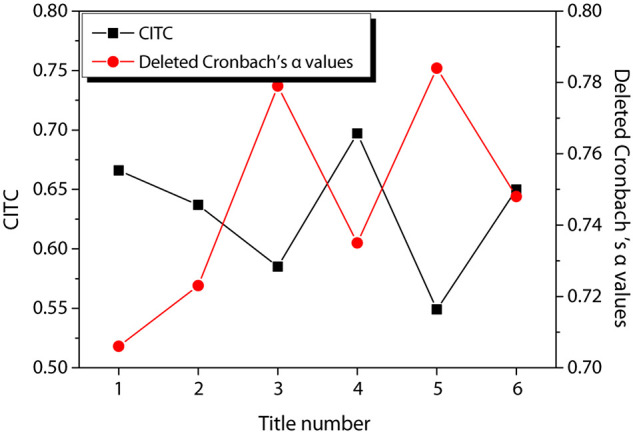
Reliability test of enterprise performance.

Figure 9 shows the reliability test results of enterprise performance. It can be observed from the Figure that the CITC values of the six items of the enterprise performance are all higher than 0.3, and their reliability coefficient values are all higher than 0.7, showing good reliability, and there is no need to delete the items for re-testing.

### Validity Test

In this study, SPSS20.0 was used for exploratory factor analysis, and KMO test statistics (Mottese et al., 2018) and Bartlett test of sphericity (Bartlett P) (Qi et al., 2019) were used as evaluation indexes to determine whether factor analysis could be carried out. Generally, the KMO value is between 0 and 1, the bigger the better, if it is less than 0.5, it means that it is not suitable for factor analysis. When the Bartlett P value is no higher than 0.01, it indicates that it is suitable for factor analysis.

Then principal component analysis (PCA) was adopted to extract factors with eigenvalues greater than 1 from the items of the four scales, and the maximum variance method was used for factor rotation. If the factor load value is lower than 0.4, the item will be deleted. If the factor load value is higher than 0.4 and the cumulative interpretation degree of the scale is higher than 50%, it means that the scale is highly valid and can be retained.

The exploratory factor analysis results of each scale are shown in Table 5. The KMO value of the entrepreneurial passion scale is greater than 0.8, and the significance probability of the Bartlett test statistical value is 0.000, indicating that it is suitable for factor analysis. The maximum variance method was adopted for factor rotation, and the eigenvalue greater than 1 is used as the factor extraction standard. Two factors are extracted, and the factor load of each item is greater than 0.5, and the cumulative explanatory degree of the scale reaches the standard of more than 50%, indicating that the scale has good validity.

**TABLE 5:** Exploratory factor analysis of each scale^a,b^.

Scale	Entrepreneurial passion		Entrepreneurial self-efficiency	Entrepreneurial persistence behavior	Enterprise performance
	Harmonious passion	Obsessive passion			
Factor load	All greater than 0.7	All greater than 0.6	All greater than 0.5	All greater than 0.7	All greater than 0.7
KMO	0.876		0.939	0.802	0.816
Bartlett test	0.000		0.000	0.000	0.000
Cumulative interpretation degree (%)	22.59	41.46	52.38	61.79	58.73

*^*a*^Principal component analysis. ^*b*^Maximum variance method.*

The KMO value of the entrepreneurial self-efficacy measurement scale is greater than 0.9, and the significance probability of the Bartlett test statistical value is 0.000, indicating that it is very suitable for factor analysis. The maximum variance method was adopted for factor rotation, and the eigenvalue greater than 1 is used as the factor extraction standard. One factor is extracted, and the factor load of each item is greater than 0.5, and the cumulative explanatory degree of the scale reaches the standard of more than 50%, indicating that the scale has good validity.

The KMO value of the entrepreneurial persistence scale is greater than 0.7, and the significance probability of the Bartlett test statistical value is 0.000, indicating that it is very suitable for factor analysis. The maximum variance method was adopted for factor rotation, and the eigenvalue greater than 1 is used as the factor extraction standard. One factor is extracted, and the factor load of each item is greater than 0.5, and the cumulative explanatory degree of the scale reaches the standard of more than 50%, indicating that the scale has good validity.

The KMO value of the enterprise performance measurement scale is greater than 0.8, and the significance probability of Bartlett test statistical value is 0.000, indicating that it is very suitable for factor analysis. The maximum variance method was adopted for factor rotation, and the eigenvalue greater than 1 is used as the factor extraction standard. One factor is extracted, and the factor load of each item is greater than 0.5, and the cumulative explanatory degree of the scale reaches the standard of more than 50%, indicating that the scale has good validity.

After exploratory factor analysis, CFA was performed according to the requirements of structural equation analysis. CFA should refer to the following three indicators: the standardized factor load value should be above 0.6, the combined reliability value should be above 0.7, and the average variable extraction value (AVE) should be above 0.5. In addition, χ^2^/df, comparative fitness index (CFI), goodness of fitness index (GFI), incremental fitness index (IFI), and the root-mean-square error of approximation (RMSEA) are used in this study for measurement. Among them, χ^2^/df is less than 3, CFI, GFI, and IFI should be greater than 0.9, and the value of RMSEA should be less than 0.08.

AMOS17.0 was used to conduct CFA on the structural validity of entrepreneurial passion scale, and the results are shown in Table 6. The standardized factor load of the two factors of entrepreneurial passion is greater than 0.6, C.R. is greater than 0.8, and AVE is greater than 0.5, indicating that the factor has a clear structure and a good scale validity. Although the value of RMSEA is 0.082, it is close to the critical value of 0.08. At the same time, the values of CFI, GFI, and IFI are all greater than the standard of 0.9, indicating a good fitting degree of entrepreneurial passion.

**TABLE 6:** CFA test of entrepreneurial passion scale.

Path	Normalized factor load	S.E.	C.R.	AVE	Goodness of fit index value
HP1 ← HP	0.758	0.074	0.884	0.558	χ^2^/df = 2.398
HP2 ← HP	0.733	0.067			CFI = 0.942
HP4 ← HP	0.739	0.062			GFI = 0.908
HP5 ← HP	0.682	0.078			IFI = 0.947
HP6 ← HP	0.749	0.069			RMSEA = 0.082
HP7 ← HP	0.806				
OP1 ← OP	0.718		0.892	0.572	
OP2 ← OP	0.738	0.098			
OP3 ← OP	0.768	0.124			
OP4 ← OP	0.658	0.126			
OP5 ← OP	0.826	0.129			
OP6 ← OP	0.831	0.146			

Table 7 shows the CFA test results of the entrepreneurial self-efficacy scale. The values of CFI, GFI, and IF are all greater than 0.9, RMSEA is 0.058, less than 0.08, and C.R. is greater than 0.7, but AVE is less than 0.5, and the standardized factor loading value of ESE14 is less than the critical value of 0.6. Therefore, the model needs to be modified to remove items with factor load lower than 0.6. The CFA test results are shown in Table 8.

**TABLE 7:** CFA test 1 of entrepreneurial self-efficacy scale.

Path	Normalized factor load	S.E.	C.R.	AVE	Goodness of fit index value
ESE1 ← ESE	0.716	0.069	0.927	0.487	χ^2^/df = 1.734
ESE2 ← ESE	0.698	0.078			CFI = 0.947
ESE3 ← ESE	0.673	0.073			GFI = 0.922
ESE4 ← ESE	0.682	0.072			IFI = 0.965
ESE5 ← ESE	0.825	0.078			RMSEA = 0.062
ESE8 ← ESE	0.682	0.083			
ESE9 ← ESE	0.679	0.094			
ESE10 ← ESE	0.714	0.082			
ESE11 ← ESE	0.632	0.093			
ESE12 ← ESE	0.636	0.086			
ESE14 ← ESE	0.572	0.088			
ESE15 ← ESE	0.791				

**TABLE 8:** CFA test 2 of entrepreneurial self-efficacy scale.

Path	Normalized factor load	S.E.	C.R.	AVE	Goodness of fit index value
ESE1 ← ESE	0.713	0.069	0.919	0.512	χ^2^/df = 1.835
ESE2 ← ESE	0.695	0.074			CFI = 0.969
ESE3 ← ESE	0.679	0.071			GFI = 0.935
ESE4 ← ESE	0.687	0.075			IFI = 0.968
ESE5 ← ESE	0.809	0.076			RMSEA = 0.057
ESE8 ← ESE	0.684	0.083			
ESE9 ← ESE	0.671	0.095			
ESE10 ← ESE	0.709	0.087			
ESE11 ← ESE	0.647	0.091			
ESE12 ← ESE	0.658	0.088			
ESE15 ← ESE	0.797				

As shown in Table 8, the values of CFI, GFI, and IFI are all greater than 0.9, and the value of RMSEA is 0.057, less than 0.08. Thus, the model has a good fitting degree. The normalized factor load values of all factors in the entrepreneurial self-efficacy scale are above 0.6, the value of C.R. is greater than 0.8, and the value of AVE is greater than 0.5, which all meet the requirements. To sum up, the measurement scale of entrepreneurial self-efficacy is effective.

Table 9 shows CFA test results of entrepreneurial persistence scale. The values of CFI, GFI, and IF are all greater than 0.9, and the value of RMSEA is 0.042 and less than 0.08, therefore, the model has a good fitting degree. The normalized factor load value of each factor in the entrepreneurial persistence behavior scale is above 0.6, the value of C.R. in the scale is greater than 0.8, and the value of AVE is greater than 0.5. To sum up, the scale of entrepreneurial persistence is effective.

**TABLE 9:** CFA test of entrepreneurial persistence behavior scale.

Path	Normalized factor load	S.E	C.R.	AVE	Goodness of fit index value
XW1 ← XW	0.738	0.153	0.802	0.501	χ^2^/df = 1.314
XW3 ← XW	0.728	0.147			CFI = 0.998
XW4 ← XW	0.679	0.141			GFI = 0.989
XW5 ← XW	0.664				IFI = 0.994 RMSEA = 0.042

Table 10 shows the CFA test results of the enterprise performance scale. The values of CFI, GFI, and IF are all greater than 0.9, and the value of RMSEA is 0.027 and less than 0.08, therefore, the model has a good fitting degree. The normalized factor load values of all factors in the enterprise performance scale are above 0.6, the value of C.R. in the scale is greater than 0.8, and the value of AVE is greater than 0.5. To sum up, the measurement scale of enterprise performance is effective.

**TABLE 10:** CFA test of enterprise performance scale.

Path	Normalized factor load	S.E.	C.R.	AVE	Goodness of fit index value
JX1 ← JX	0.779	0.162	0.831	0.557	χ^2^/df = 1.379
JX2 ← JX	0.768	0.151			CFI = 0.944
JX3 ← JX	0.680	0.147			GFI = 0.991
JX5 ← JX	0.689				IFI = 0.993 RMSEA = 0.027

### Correlation Analysis

In order to test whether there is a correlation between the variables, the Pearson correlation coefficient was adopted for the test. The results are shown in Table 11. It can be observed from the data in the Table 11 that there is a good correlation between the variables, and there is no collinearity. Harmonious passion and obsessive passion have a significant positive relationship with entrepreneurial persistence behavior and enterprise performance, indicating that entrepreneurial passion has a positive impact on entrepreneurial behavior and enterprise performance. Harmonious passion and obsessive passion are significantly correlated with entrepreneurial self-efficacy, indicating that entrepreneurial passion has a positive impact on self-efficacy, and meets the relationship model of entrepreneurial passion, entrepreneurial self-efficacy, entrepreneurial persistence, and enterprise performance designed in this study.

**TABLE 11:** Correlation coefficient between variables.

Variables	Mean	Standard deviation	1	2	3	4	5
Harmonious passion	4.191	0.465	1				
Obsessive passion	3.587	0.726	0.306**	1			
Entrepreneurial self-efficacy	3.891	0.578	0.467**	0.143*	1		
Entrepreneurial persistence behavior	3.784	0.496	0.452**	0.536**	0.245**	1	
Enterprise performance	3.508	0.842	0.483**	0.503**	0.478**	0.609**	1

*Indicates a significant correlation at 0.05; and **indicates a significant correlation at 0.01.

## Structural Equation Simulation Analysis

In this study, χ^2^ values, χ^2^/df, GFI, CFI, IFI, and RMSEA were selected to evaluate the adaptability of the model. According to the theoretical model of the influence mechanism of entrepreneurial passion and entrepreneurial self-efficacy on entrepreneurial persistence behavior and enterprise performance and the conclusion of correlation analysis proposed in this study, the corresponding path analysis of the relationship between variables was performed in AMOS20.0, as shown in Table 12. The ratio of model CMIN to degree of freedom is less than 5, RMSEA is less than 0.08, but GFI and IFI have not reached the critical value of 0.9. Although the difference between these values and the ideal standard values is small, the adaptability of the model is not high. Therefore, the model should be further modified to improve the fitting degree of the model.

**TABLE 12:** Initial model analysis results.

Hypothesis path	Estimate	S.E.	C.R.	*P*
Self-efficacy ← harmonious passion	0.406	0.074	5.499	***
Self-efficacy ← compulsive passion	0.325	0.068	4.773	***
Entrepreneurial persistence behavior ← self-efficacy	0.447	0.079	5.696	***
Entrepreneurial persistence behavior ← harmonious passion	0.112	0.076	1.478	0.139
Entrepreneurial persistence behavior ← compulsive passion	0.285	0.071	4.024	***
Enterprise performance ← self-efficacy	0.426	0.052	1.929	***
Enterprise performance ← harmonious passion	0.188	0.086	1.625	0.156
Enterprise performance ← compulsive passion	0.171	0.056	1.376	***
Fitting index	χ^2^ = 764.430; df = 582; χ^2^/df = 1.313; GFI = 0.836; CFI = 0.949; IFI = 0.819; RMSEA = 0.039			

***Indicates a significant correlation at 0.001.

According to the MI correction index principle, the relationship between the residuals is increased from large to small according to the value of MI and tested. It is found that the fitting indexes of the model are improved. The value χ^2^/df is less than 3, the value of CFI is more than 0.9, and the value of RMSEA is less than 0.08 of the model. Although the values of GFI and IFI do not reach the critical value of 0.9, the difference from the critical value is small, and the other standards all meet the requirements. Therefore, it is believed the fitting degree between the sample data and the model is good. The model fitting results are shown in Table 13.

**TABLE 13:** Model analysis results after revision.

Hypothesis path	Estimate	S.E.	C.R.	*P*
Self-efficacy ← harmonious passion	0.335	0.082	5.165	***
Self-efficacy ← compulsive passion	0.392	0.068	4.507	***
Entrepreneurial persistence behavior ← self-efficacy	0.497	0.083	5.499	***
Entrepreneurial persistence behavior ← harmonious passion	0.392	0.077	1.679	0.093
Entrepreneurial persistence behavior ← compulsive passion	0.328	0.066	4.203	***
Enterprise performance ← self-efficacy	0.489	0.054	1.951	***
Enterprise performance ← harmonious passion	0.192	0.085	1.636	0.051
Enterprise performance ← compulsive passion	0.182	0.058	1.378	***
Fitting index	χ^2^ = 652.020; df = 577; χ^2^/df = 1.131; GFI = 0.862; CFI = 0.978; IFI = 0.851; RMSEA = 0.024			

***Indicates a significant correlation at 0.001.

As shown in Tables 12, 13, compulsive passion has a significant direct effect on entrepreneurial persistence and corporate performance, while HP has a positive relationship with entrepreneurial persistence and corporate performance, but the direct effect is not significant. Therefore, H1 has been partially verified that the direct effect of HP on entrepreneurial persistence and enterprise performance is not significant, while the direct effect of compulsive passion on entrepreneurial persistence and enterprise performance is significant.

Both HP and compulsive passion have significant positive effects on entrepreneurial self-efficacy, indicating that the higher the entrepreneurial passion is, the higher the self-efficacy is, and the more confident they are in their entrepreneurial ability. Therefore, H2 has been verified.

Entrepreneurial self-efficacy has a significant positive impact on entrepreneurial persistence and corporate performance, so H3 is valid.

The direct effects of HP and compulsive passion on entrepreneurial self-efficacy are significant, while the direct effects of entrepreneurial self-efficacy on entrepreneurial persistence and enterprise performance are significant, and the direct effects of HP and compulsive passion on entrepreneurial persistence and enterprise performance are significant. Therefore, entrepreneurial self-efficacy plays a significant mediating role between HP, compulsive passion, entrepreneurial persistence, and enterprise performance, and H4 has been verified.

Most of the hypotheses and theoretical models proposed in this study have been verified. Although the hypotheses are supported by the relevant empirical studies and theories of foreign scholars, due to the different social environment in China and the difference in data quality in the process of questionnaire collection, some hypotheses are valid. Table 14 is the summary of hypothesis verification obtained by the empirical research in this study.

**TABLE 14:** Summary of hypothesis validation.

Item	Hypothesis	Result
H1	Entrepreneurial passion has positive influence on entrepreneurial persistence and enterprise performance	Partially valid
H1-1	Harmonious passion has positive influence on entrepreneurial persistence and enterprise performance	Invalid
H1-2	Compulsive passion has positive influence on entrepreneurial persistence and enterprise performance	Valid
H2	Entrepreneurial passion has a positive effect on self-efficacy	Valid
H2-1	Harmonious passion has positive influence on self-efficacy	Valid
H2-2	Compulsive passion has a positive effect on self-efficacy	Valid
H3	Self-efficacy has positive influence on entrepreneurial persistence behavior and enterprise performance	Valid
H4	Self-efficacy plays an intermediary role between entrepreneurial passion, entrepreneurial persistence, and corporate performance	Valid
H4-1	Self-efficacy plays an intermediary role between harmonious passion, entrepreneurial persistence, and enterprise performance	Valid
H4-2	Self-efficacy mediates between compulsive passion, entrepreneurial persistence, and corporate performance	Valid

## Discussion

In order to study the influence of entrepreneurial passion on the psychology and behavior of entrepreneurs, in this research, based on the self-efficacy theory, the relationship model between entrepreneurial passion and the psychology and behavior of entrepreneurs was built. The relationship between entrepreneurial passion, self-efficacy, entrepreneurial persistence behavior, and enterprise performance was analyzed through questionnaire survey, the reliability and validity of the questionnaire were tested, and the correlation among variables was discussed. The results show that the designed questionnaire has a high reliability and validity, and the self-efficacy plays an intermediary role between the entrepreneurial passion and the entrepreneur’s psychology and behavior (Warnick et al., 2018). The entrepreneurial passion can positively guide the entrepreneur’s entrepreneurial persistence behavior, and at the same time, it can stimulate the entrepreneur’s positive emotion and promote the enterprise’s performance (Wiklund et al., 2018). For entrepreneurs, they can improve their entrepreneurial role recognition, maintain an optimistic and positive attitude, stimulate creativity and innovation power, and enhance their sense of self-efficacy. The government can also promote successful entrepreneurship cases, establish a social environment that is inclusive of innovation, and stimulate the entrepreneurial passion of entrepreneurs (Dillen et al., 2019). Warnick et al. (2018) believe that both product enthusiasm and entrepreneurial enthusiasm have a certain impact on venture capital. When investors believe that entrepreneurs are highly open and willing to accept feedback, both kinds of passion will become more attractive, which indicates that openness to feedback alleviates potential concerns related to passion. Meanwhile, venture investors have different considerations on passion. Angel investors and venture capitalists with more investment experience pay more attention to the combination of product enthusiasm and open feedback. The results also verify the influence mechanism of entrepreneurial passion on entrepreneurial behavior in this study, which is consistent with the hypothesis proposed in this study. Klyver et al. (2018) also studied the timing of social support, that was, how entrepreneurial passion (emotional support and tool support) affected the entrepreneurial perseverance of new entrepreneurs. Through the test over the hypotheses through the longitudinal data set of emerging entrepreneurs, it was found that emotional support was most relevant at the early stage of venture development of entrepreneurial projects, while instrumental support was most relevant to entrepreneurs who started their businesses at the early stage (Klyver et al., 2018), which also supported the results of this study. Idkhan and Idris (2018) studied the causal structure among entrepreneur knowledge (X1), self-efficacy (X2), and entrepreneurial intention (Y). Data were collected through questionnaire survey, and descriptive and path analysis were used to decompose the data. The results showed that X1 and X2 had a significant influence on Y (Idkhan and Idris, 2018). The results further illustrated the influence mechanism of self-efficacy on entrepreneur’s persistence behavior, which is the same as the results of this study.

## Conclusion

In order to study the influence of entrepreneurial passion on entrepreneurs’ psychology and behavior, based on the self-efficacy theory, the relationship model between entrepreneurial passion and entrepreneurs’ psychology and behavior was constructed. The relationship between entrepreneurial passion, self-efficacy, entrepreneurial persistence, and enterprise performance was analyzed through questionnaire survey. Through exploratory factor analysis and CFA, it is found that the designed questionnaire has higher reliability and validity. The results of structural equation simulation show that the direct effect of HP on entrepreneurial persistence and enterprise performance is not significant, while the direct effect of compulsive passion on entrepreneurial persistence and enterprise performance is significant. Harmonious passion and compulsive passion are significantly correlated with entrepreneurial self-efficacy, and self-efficacy plays an intermediary role between entrepreneurial passion and entrepreneurs’ psychology and behavior (Zhu et al., 2019). The hypothesis proposed is basically valid. This study reveals the relationship between entrepreneurial passion, self-efficacy, psychology and behavior of entrepreneurs, and expands the application of entrepreneurial passion in the field of entrepreneurship. However, the research method of questionnaire adopted may lead to the subjective bias of the respondents, and the accuracy of the data decreases. In future studies, the performance of entrepreneurs can be evaluated by others to improve the reliability and authenticity of the data, and the reliability of the results can also be improved by increasing the sample size.

## Data Availability Statement

The raw data supporting the conclusions of this article will be made available by the authors, without undue reservation.

## Ethics Statement

The studies involving human participants were reviewed and approved by the Northwest University Ethics Committee. The patients/participants provided their written informed consent to participate in this study.

## Author Contributions

Both authors listed have made a substantial, direct and intellectual contribution to the work, and approved it for publication.

## Conflict of Interest

The authors declare that the research was conducted in the absence of any commercial or financial relationships that could be construed as a potential conflict of interest.
